# Machine learning-based prediction model for 30-day readmission risk in elderly patients with type 2 diabetes mellitus and heart failure: a retrospective cohort study with SHAP interpretability analysis

**DOI:** 10.3389/fcvm.2025.1673159

**Published:** 2026-01-07

**Authors:** Lei Wang, Chao Wei, Yue Chen, Da-Sheng Lu, Hong-Xiang Zhang, Mo Yang

**Affiliations:** Department of Cardiovascular Medicine, The Second Affiliated Hospital of Wannan Medical College, Wuhu, Anhui, China

**Keywords:** type 2 diabetes mellitus, heart failure, 30-day readmission, machine learning, risk prediction

## Abstract

**Objective:**

Among elderly populations with concurrent type 2 diabetes mellitus (T2DM) and heart failure (HF), 30-day hospital readmission rates range 10%–25%. Conventional risk evaluation instruments show restricted predictive performance (AUC < 0.70) in this multimorbid group. This research aimed to construct and verify an artificial intelligence-based algorithm for assessing 30-day readmission probability in elderly T2DM-HF patients.

**Methods:**

This retrospective cohort study included 870 participants ≥65 years with T2DM and HF (January 2020–December 2023), randomly divided into training (*n* = 609, 70%) and validation (*n* = 261, 30%) cohorts. Variable selection utilized Least Absolute Shrinkage and Selection Operator with ten-fold cross-validation. Eight machine learning algorithms were evaluated: logistic regression, random forest, gradient boosting machines, support vector machines, neural networks, convolutional neural networks, AdaBoost, and stacking ensemble. Model interpretability was enhanced using SHapley Additive exPlanations analysis.

**Results:**

Overall 30-day readmission rate was 12.4% (108/870 patients). The Stacking Ensemble model achieved superior performance with AUC 0.867 (95% CI: 0.830–0.904), accuracy 79.4%, sensitivity 74.9%, and specificity 84.0%. Fourteen key predictors were identified, with C-reactive protein, estimated glomerular filtration rate, and B-type natriuretic peptide as most influential factors.

**Conclusion:**

This study developed a high-performing, interpretable machine learning model for predicting 30-day readmission risk, providing a valuable clinical decision-making tool.

## Introduction

Type 2 diabetes mellitus (T2DM) and heart failure (HF) frequently coexist in elderly patients, creating complex clinical scenarios with significant healthcare implications. Globally, T2DM impacts more than 537 million adult individuals, showing peak occurrence rates among those 65 years and above, whereas HF influences roughly 64 million persons worldwide (1). The bidirectional relationship between these conditions is well-established, with diabetes increasing heart failure risk by 2−4 fold (2, 3).

Thirty-day hospital readmissions constitute a vital quality metric and significant financial burden, generating worldwide healthcare expenditures amounting to billions of dollars per year (4). Among elderly patients with comorbid T2DM and HF, 30-day readmission rates range from 10% to 25%, significantly higher than younger populations or single-condition patients (5–7). Healthcare systems worldwide have implemented quality improvement initiatives targeting readmission reduction, emphasizing the need for accurate risk prediction tools (8).

Conventional risk evaluation instruments exhibit constrained precision in aged patients with numerous comorbid conditions, generally attaining receiver operating characteristic curve values under 0.70 (9, 10). These limitations stem from inadequate capture of complex interactions between age-related changes, polypharmacy, and social determinants of health. Machine learning approaches have shown superior performance by analyzing high-dimensional datasets and identifying non-linear relationships, with ensemble methods achieving significantly higher accuracy than conventional statistical methods (11, 12). The integration of explainable artificial intelligence, particularly SHapley Additive exPlanations (SHAP), has become crucial for clinical acceptance and understanding of model predictions (13, 14).

Despite advances in predictive modeling, specialized tools for elderly patients with T2DM and HF remain limited, with most existing models focusing on single conditions or general populations (15, 16). Therefore, this study aimed to develop and validate a comprehensive machine learning model for predicting 30-day readmission risk in elderly patients with T2DM complicated by heart failure, incorporating multiple algorithmic approaches and SHAP-based interpretability analysis to create a clinically applicable tool for early identification of high-risk patients.

## Methods

### Study design and participants

This monocentric historical cohort investigation was structured to construct and internally verify a clinical prognostic algorithm for 30-day readmission probability in aged patients with T2DM accompanied by HF. Electronic health record information was gathered from the medical facility spanning January 2020 through December 2023. This single-center design was chosen to ensure data quality and consistency through standardized electronic health record systems and complete follow-up protocols established at our institution.

Participant inclusion required satisfaction of these conditions: chronological age of 65 years or greater, verified T2DM diagnosis based on World Health Organization (WHO) or American Diabetes Association (ADA) standards, concurrent diagnosis of HF meeting the 2021 European Society of Cardiology (ESC) heart failure guidelines, comprehensive clinical information encompassing demographic details and laboratory findings, and medical facility stay duration of 24 h or more. Participants were eliminated if they presented with type 1 diabetes or alternative specific diabetes forms, acute coronary events or acute cerebrovascular incidents, malignant neoplasms, severe hepatic dysfunction (Child-Pugh C grade) or end-stage renal disease requiring dialysis, in-hospital death, missing key clinical data exceeding 20%, or transfer to other hospitals or self-discharge.

A total of 870 eligible patients were ultimately included and randomly divided into training (609 patients, 70%) and validation (261 patients, 30%) cohorts using stratified random sampling.

Ethical approval for this investigation was obtained from the Institutional Review Board of the Second Affiliated Hospital of Wannan Medical College, with all procedures adhering to the principles outlined in the Declaration of Helsinki. Due to the retrospective study design and utilization of anonymized datasets, the requirement for informed consent was exempted

### Data collection and variable definition

Clinical data were systematically extracted from electronic medical records and laboratory information systems. Heart failure was classified according to left ventricular ejection fraction (LVEF) as heart failure with reduced ejection fraction (HFrEF, LVEF <40%), heart failure with mildly reduced ejection fraction (HFmrEF, LVEF 40%–49%), and heart failure with preserved ejection fraction (HFpEF, LVEF ≥50%) based on echocardiographic assessments performed during hospitalization. Candidate variables included demographic characteristics [age, gender, body mass index (BMI), length of hospital stay, previous hospitalizations, surgical history, smoking history, alcohol consumption history], comorbidities (hypertension, coronary heart disease, cerebral infarction, hyperlipidemia, chronic gastritis, osteoporosis, pulmonary infection, chronic kidney disease, atrial fibrillation or other arrhythmias, chronic obstructive pulmonary disease, anemia, peripheral vascular disease, depression/anxiety status), medication usage [statin use, antiplatelet/anticoagulant use, insulin use, oral hypoglycemic drug use, angiotensin-converting enzyme inhibitor/angiotensin receptor blocker (ACEI/ARB) use, β-blocker use, diuretic use], and laboratory parameters (lipid profiles, renal function markers, glycemic control indicators, cardiac biomarkers, inflammatory markers, nutritional markers, electrolytes, endocrine markers), and cardiac function indicators (LVEF, heart failure classification).

### Outcome definition

The principal endpoint constituted 30-day readmission, characterized as any unscheduled hospital re-entry occurring within 30 days following discharge regardless of underlying cause. Planned admissions including elective surgery, rehabilitation therapy, nursing home transfers, and other scheduled hospitalizations were excluded. Hospital transfers within the same admission period were treated as single hospitalization episodes, while transfers between different admission periods were classified as separate readmissions. Readmission status was confirmed through hospital information system records supplemented by telephone follow-up when necessary. Patients who died within 30 days post-discharge were identified through hospital mortality databases and death registry linkage. These patients were censored at the time of death and classified as competing events rather than readmissions, as death precludes the possibility of hospital readmission. The 30-day mortality rate was reported separately as a secondary outcome.

### Feature selection and model development

Data preprocessing involved multiple steps to ensure quality and statistical validity. Missing values were handled using multiple imputation with chained equations for variables with missing rates between 5% and 20%, while variables with missing rates exceeding 20% were excluded. Continuous variables were standardized using *Z*-score transformation to ensure comparability across different measurement scales.

Feature selection employed Least Absolute Shrinkage and Selection Operator (LASSO) regression with 10-fold cross-validation to identify the optimal subset of predictors while preventing overfitting. Candidate variables included demographic characteristics, comorbidities, medication usage, and laboratory parameters. Univariate screening (*P* < 0.20) was performed prior to LASSO to retain potentially relevant predictors. The LASSO algorithm used coordinate descent optimization with elastic net mixing parameter *α* = 1. The regularization parameter *λ* was tuned across logarithmically-spaced values from *λ*_max to *λ*_min. The regularization parameter *λ* was selected by minimizing cross-validation error using the one-standard-error rule. Bootstrap validation with 1,000 resamples confirmed feature selection stability (variables selected in >80% of samples). Variance inflation factors (VIF) were calculated for selected variables to assess multicollinearity, with values <5 indicating absence of significant collinearity.

Eight machine learning algorithms were systematically evaluated: logistic regression, random forest (RF), gradient boosting machine (GBM), support vector machine (SVM), neural network, convolutional neural network (CNN), AdaBoost, and stacking ensemble. The inclusion of CNN in our comparative analysis, despite its primary application in image processing, was motivated by recent studies demonstrating its potential for capturing complex non-linear patterns in structured tabular data. For CNN implementation, the 14 selected features were structured as a 1-dimensional input vector and processed through one-dimensional convolutional layers. This architectural choice was based on the hypothesis that CNN's ability to learn hierarchical feature representations and local patterns through convolutional filters might reveal meaningful interactions between clinical and laboratory parameters that traditional tabular models might miss. The CNN architecture consisted of three 1D convolutional layers (32, 64, and 128 filters respectively) with kernel size of 3, followed by max pooling layers, dropout layers for regularization (dropout rate = 0.3), and fully connected layers for final classification. This approach allowed us to explore whether spatial/sequential relationships among the ordered features (e.g., inflammatory markers, cardiac biomarkers, renal function parameters) could enhance predictive performance compared to conventional machine learning methods that treat features as independent variables. Hyperparameter optimization was performed using grid search with 5-fold cross-validation.

### Model validation and evaluation

Model performance was comprehensively evaluated using multiple metrics. Discriminative ability was assessed using receiver operating characteristic curves (ROC) and area under the curve (AUC), along with sensitivity, specificity, positive predictive value (PPV), negative predictive value (NPV), accuracy, and F1-score. Model calibration was evaluated using calibration plots with 1,000 bootstrap resamples. Clinical utility was assessed through decision curve analysis to determine net clinical benefit across different threshold probabilities, with clinical impact curves generated to visualize estimated numbers of high-risk patients at various risk thresholds.

Model interpretability was enhanced using SHAP analysis to quantify individual feature contributions to prediction outcomes. SHAP values were calculated to create feature importance rankings, summary plots, and waterfall plots for individual predictions, providing clinicians with transparent insights into the decision-making process.

### Clinical decision support tool development

Based on optimal model performance, a user-friendly web-based clinical risk calculator was developed to facilitate point-of-care decision-making. The calculator incorporated all selected features and provided real-time risk assessment with visual risk gauges. The tool included input validation mechanisms and immediate feedback capabilities, enabling appropriate intervention planning for high-risk patients. Protocols for ongoing model monitoring and updating were established, including regular performance evaluations and strategies for detecting population drift.

### Statistical analysis

Categorical variables are expressed as counts and proportions, with comparisons performed via chi-square testing or Fisher's exact testing when applicable. Continuous variables are displayed as arithmetic mean ± standard deviation (SD) or median accompanied by interquartile range (IQR), with comparisons executed using Student's *t*-testing or Mann–Whitney *U* testing depending on distributional characteristics. All statistical computations were conducted utilizing Python version 3.9 and R version 4.3.0 platforms. Artificial intelligence algorithms were developed using scikit-learn, XGBoost, and LightGBM frameworks. SHAP evaluation was performed employing the shap library. Statistical significance was established at *α* = 0.05 for bilateral testing, with all confidence intervals (CI) computed at the 95% confidence level. Thirty-day all-cause mortality was documented as a secondary outcome. Patients who died during the follow-up period were censored at the time of death for readmission analysis.

## Results

### Patient characteristics

A total of 870 elderly T2DM patients complicated with heart failure were included in this study and randomly allocated into training (*N* = 609, 70%) and validation (*N* = 261, 30%) cohorts. Participant demographics and clinical features are outlined in Table 1. The median age was 76.0 years (IQR: 71.0–81.0), with females comprising 488 patients (56.1%) of the total population. The mean BMI was 24.6 ± 3.8 kg/m^2^, and the median length of hospital stay was 8.0 days (IQR: 5.0–12.0 days).

**Table 1:** Baseline characteristics comparison of elderly T2DM patients with heart failure.

Variable	Total Sample (*N* = 870)	Training Group (*N* = 609)	Validation Group (*N* = 261)	*P*-value
Gender				0.177
Male	382 (43.9)	273 (44.8)	109 (41.8)	–
Female	488 (56.1)	336 (55.2)	152 (58.2)	–
BMI, kg/m^2^	24.6 ± 3.8	24.5 ± 3.7	24.8 ± 4.0	0.335
Previous surgical history	573 (65.9)	402 (66.0)	171 (65.5)	0.885
Smoking history	269 (30.9)	189 (31.0)	80 (30.7)	0.912
Alcohol consumption history	204 (23.4)	145 (23.8)	59 (22.6)	0.692
Length of hospital stay	8.0 (5.0–12.0)	8.0 (5.0–12.0)	8.0 (5.0–11.0)	0.464
Previous hospitalizations	2.0 (1.0–3.0)	2.0 (1.0–3.0)	2.0 (1.0–3.0)	0.852
Gender				0.177
Male	382 (43.9)	273 (44.8)	109 (41.8)	–
Female	488 (56.1)	336 (55.2)	152 (58.2)	–
BMI	24.6 ± 3.8	24.5 ± 3.7	24.8 ± 4.0	0.335
Previous surgical history	573 (65.9)	402 (66.0)	171 (65.5)	0.885
Smoking history	269 (30.9)	189 (31.0)	80 (30.7)	0.912
Alcohol consumption history	204 (23.4)	145 (23.8)	59 (22.6)	0.692
Length of hospital stay, days	8.0 (5.0–12.0)	8.0 (5.0–12.0)	8.0 (5.0–11.0)	0.464
Previous hospitalizations, times	2.0 (1.0–3.0)	2.0 (1.0–3.0)	2.0 (1.0–3.0)	0.852
Hypertension	736 (84.6)	516 (84.7)	220 (84.3)	0.885
Coronary heart disease	616 (70.8)	431 (70.8)	185 (70.9)	0.976
Cerebral infarction	202 (23.2)	141 (23.2)	61 (23.4)	0.941
Hyperlipidemia	176 (20.2)	119 (19.5)	57 (21.8)	0.431
Chronic gastritis	105 (12.1)	72 (11.8)	33 (12.6)	0.727
Osteoporosis	124 (14.3)	87 (14.3)	37 (14.2)	0.975
Pulmonary infection	95 (10.9)	68 (11.2)	27 (10.3)	0.692
Chronic kidney disease	278 (32.0)	195 (32.0)	83 (31.8)	0.948
Atrial fibrillation or other arrhythmias	217 (24.9)	152 (25.0)	65 (24.9)	0.978
Chronic obstructive pulmonary disease	139 (16.0)	97 (15.9)	42 (16.1)	0.942
Anemia	313 (36.0)	219 (36.0)	94 (36.0)	1.000
Peripheral vascular disease	87 (10.0)	61 (10.0)	26 (10.0)	1.000
Depression and anxiety	78 (9.0)	54 (8.9)	24 (9.2)	0.875
Statin use	612 (70.3)	428 (70.3)	184 (70.5)	0.955
Antiplatelet/anticoagulant use	717 (82.4)	502 (82.4)	215 (82.4)	1.000
Insulin use	522 (60.0)	365 (59.9)	157 (60.2)	0.938
Oral hypoglycemic drug use	696 (80.0)	487 (80.0)	209 (80.1)	0.967
ACEI/ARB use	643 (73.9)	450 (73.9)	193 (73.9)	1.000
β-blocker use	574 (66.0)	402 (66.0)	172 (65.9)	0.978
Diuretic use	435 (50.0)	304 (49.9)	131 (50.2)	0.938
Triglycerides, mmol/L	1.39 (1.00–1.99)	1.37 (0.99–2.02)	1.43 (1.02–1.94)	0.648
Creatinine, μmol/L	78.6 (62.97–100.20)	79.3 (64.20–101.45)	76.3 (60.10–98.50)	0.068
Uric acid, μmol/L	350.2 (284.6–422.0)	348.2 (285.3–426.5)	352.0 (282.9–411.9)	0.587
LDL cholesterol, mmol/L	2.35 ± 0.89	2.34 ± 0.88	2.37 ± 0.91	0.648
HDL cholesterol, mmol/L	1.08 ± 0.32	1.07 ± 0.31	1.09 ± 0.34	0.434
HbA1c, %	7.20 (6.50–8.40)	7.20 (6.40–8.30)	7.30 (6.50–8.60)	0.815
Fasting glucose, mmol/L	7.66 (5.97–10.63)	7.66 (5.97–10.72)	7.65 (6.01–10.40)	0.902
eGFR, mL/min/1.73 m^2^	76.5 (55.0–94.3)	76.0 (53.7–93.4)	77.0 (57.1–98.5)	0.373
Neutrophil-to-albumin ratio	17.59 (15.35–20.20)	17.69 (15.37–20.36)	17.25 (15.29–19.77)	0.217
Hemoglobin, g/L	119.5 ± 18.3	119.2 ± 18.1	120.1 ± 18.7	0.571
Serum albumin, g/L	36.8 ± 4.2	36.7 ± 4.1	37.0 ± 4.4	0.373
BNP/NT-proBNP, pg/mL	1,285.5 (658.3–2,456.8)	1,298.2 (672.1–2,489.3)	1,256.7 (635.4–2,398.1)	0.498
C-reactive protein, mg/L	8.2 (3.1–18.5)	8.3 (3.2–18.9)	7.9 (2.9–17.8)	0.666
Serum sodium, mmol/L	139.2 ± 3.8	139.1 ± 3.7	139.4 ± 4.0	0.373
Serum potassium, mmol/L	4.12 ± 0.48	4.11 ± 0.47	4.15 ± 0.50	0.324
TSH, mIU/L	2.18 (1.35–3.42)	2.21 (1.38–3.45)	2.12 (1.29–3.35)	0.587
25-hydroxyvitamin D, ng/mL	18.3 (12.1–26.8)	18.5 (12.3–27.1)	17.9 (11.8–26.2)	0.498
30-day readmission	108 (12.4)	76 (12.5)	32 (12.3)	0.933

T2DM, type 2 diabetes mellitus; BMI, body mass index; ACEI/ARB, angiotensin-converting enzyme inhibitor/angiotensin receptor blocker; LDL, low-density lipoprotein; HDL, high-density lipoprotein; HbA1c, hemoglobin A1c (glycated hemoglobin); eGFR, estimated glomerular filtration rate; BNP/NT-proBNP, B-type natriuretic peptide/N-terminal pro-B-type natriuretic peptide; TSH, thyroid stimulating hormone.

Categorical variables are presented as *n* (%); continuous variables with normal distribution are presented as mean ± standard deviation; continuous variables with non-normal distribution are presented as median (interquartile range); *P*-values calculated using chi-square test for categorical variables and *t*-test or Mann–Whitney *U* test for continuous variables.

No statistically significant differences were observed between the training and validation groups across all baseline variables (all *P* > 0.05), indicating successful randomization and comparable cohort characteristics. Principal comorbid conditions encompassed hypertension in 736 participants (84.6%), coronary artery disease in 616 participants (70.8%), chronic renal disease in 278 participants (32.0%), and anemia in 313 participants (36.0%). The prevalence of pulmonary infections was 95 patients (10.9%), which emerged as a significant predictor in subsequent analyses.

Regarding medication utilization, 696 patients (80.0%) were receiving oral hypoglycemic drugs, 643 patients (73.9%) were on ACEI/ARB therapy, 574 patients (66.0%) were prescribed β-blockers, and 435 patients (50.0%) were using diuretics. Laboratory findings revealed median concentrations for essential biomarkers: glycated hemoglobin (HbA1c) of 7.20% (IQR: 6.50%–8.40%), estimated glomerular filtration rate (eGFR) of 76.5 mL/min/1.73 m^2^ (IQR: 55.0–94.3), B-type natriuretic peptide/N-terminal pro-B-type natriuretic peptide (BNP/NT-proBNP) of 1,285.5 pg/mL (IQR: 658.3–2,456.8), C-reactive protein (CRP) of 8.2 mg/L (IQR: 3.1–18.5), and neutrophil-to-albumin ratio of 17.59 (IQR: 15.35–20.20). The overall 30-day readmission rate was 12.4% (108/870 patients), with 12.5% (76/609) in the training group and 12.3% (32/261) in the validation group (*P* = 0.933), demonstrating no significant difference in outcome distribution between cohorts. Regarding heart failure classification based on LVEF, 412 patients (47.4%) had HFrEF (LVEF <40%), 198 patients (22.8%) had HFmrEF (LVEF 40%–49%), and 260 patients (29.9%) had HFpEF (LVEF ≥50%). The distribution of heart failure subtypes was comparable between training and validation cohorts (*P* = 0.876). The mean LVEF was 42.3 ± 11.8% in the overall cohort, with no significant difference between readmitted and non-readmitted patients (41.8 ± 12.1% vs. 42.4 ± 11.7%, *P* = 0.582).

During the 30-day follow-up period, 23 patients (2.6%) died without prior readmission: 17 (2.8%) in the training cohort and 6 (2.3%) in the validation cohort (*P* = 0.682). These deaths were treated as competing events in the analysis. The most common causes of death were cardiac-related events (*n* = 12, 52.2%), followed by respiratory failure (*n* = 6, 26.1%) and multi-organ failure (*n* = 5, 21.7%). When accounting for the competing risk of death, the cumulative incidence of readmission remained consistent with the overall rate.

### Feature selection and variable importance

**Figure 1:**
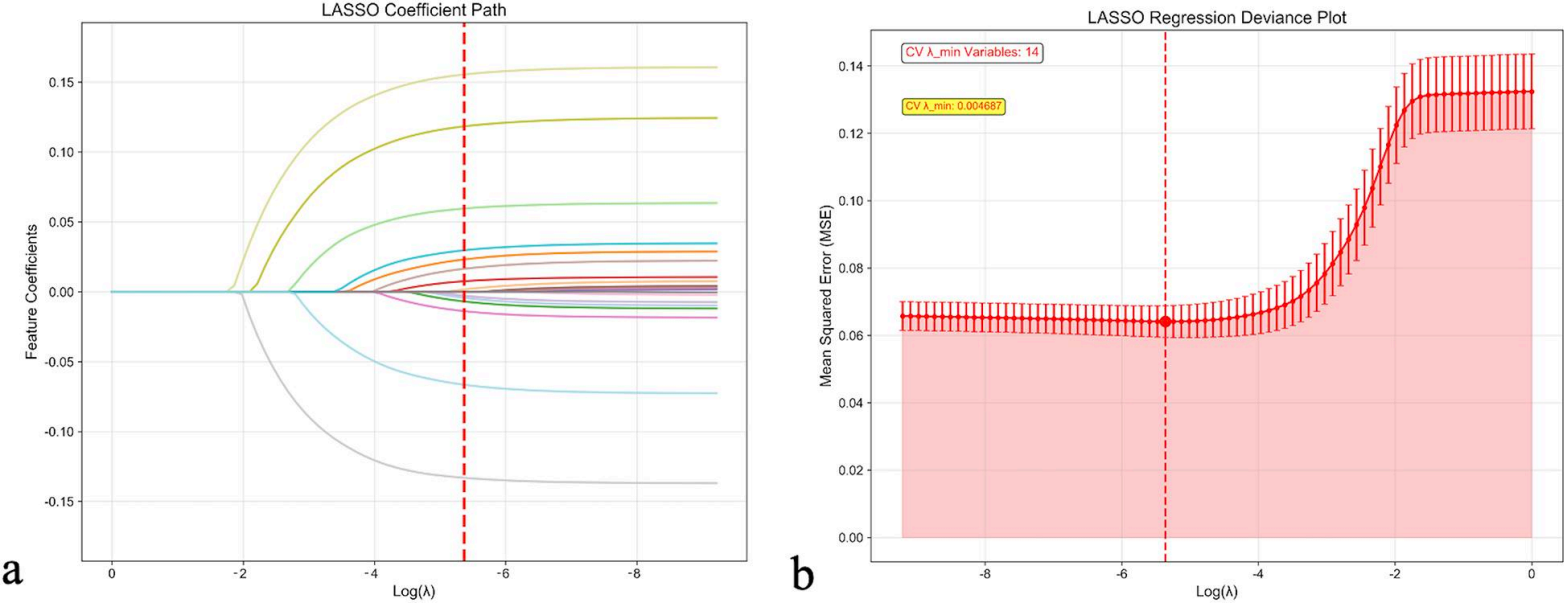
LASSO regression feature selection analysis. **(a)** LASSO coefficient path showing the shrinkage behavior of feature coefficients as the regularization parameter (*λ*) increases. Each colored line represents a different feature, with coefficients approaching zero as *λ* increases. The red dashed line indicates the optimal *λ* value selected by cross-validation. **(b)** LASSO regression deviance plot displaying the cross-validation error (Mean Squared Error) as a function of log (*λ*). The optimal *λ* value (*λ*_min = 0.004687) that minimizes cross-validation error is marked by the red dashed line, resulting in the selection of 14 key variables. Error bars represent standard errors across cross-validation folds. CV, cross-validation; LASSO, least absolute shrinkage and selection operator.

Feature selection was conducted using least absolute shrinkage and selection operator (LASSO) regression with 10-fold cross-validation to identify the optimal subset of predictors while preventing overfitting. As demonstrated in Figure 1, the LASSO coefficient path illustrated the shrinkage behavior of individual variables as the regularization parameter (*λ*) increased. Cross-validation identified the optimal *λ* value (*λ*_min = 0.004687) that minimized prediction error, resulting in the selection of 14 key variables for the final prediction model.

The selected features included clinical, laboratory, and treatment-related variables: CRP, eGFR, BNP/NT-proBNP, hemoglobin levels, length of hospital stay, HbA1c, BMI, anemia status, diuretic use, previous hospitalizations, alcohol consumption history, age, chronic kidney disease, and smoking history. The VIF for all selected variables were <5, indicating absence of significant multicollinearity among predictors.

### Model development and performance comparison

Eight different machine learning algorithms were systematically evaluated and compared for predicting 30-day readmission risk, as detailed in Table 2. In the training cohort, the Neural Network model demonstrated the highest discriminative performance with an AUC of 0.990 (95% CI: 0.985–0.995), followed by the CNN model (AUC: 0.913, 95% CI: 0.890–0.936) and Stacking Ensemble (AUC: 0.895, 95% CI: 0.870–0.920). However, when evaluating generalizability to independent datasets, the Stacking Ensemble model exhibited superior and more stable performance across validation cohorts.

**Table 2:** Model performance comparison.

Model	AUC	Accuracy	Sensitivity	Specificity	PPV	NPV	F1-score
Training Group							
Logistic Regression	0.711	0.635	0.826	0.445	0.598	0.718	0.694
Random Forest	0.759	0.699	0.694	0.703	0.701	0.697	0.697
Gradient Boosting	0.887	0.802	0.76	0.845	0.83	0.779	0.794
SVM	0.856	0.77	0.865	0.675	0.727	0.833	0.79
Neural Network	0.99	0.856	0.992	0.721	0.78	0.989	0.874
CNN	0.913	0.829	0.898	0.759	0.788	0.882	0.84
AdaBoost	0.751	0.689	0.683	0.695	0.691	0.687	0.687
Stacking Ensemble	0.895	0.809	0.768	0.849	0.836	0.786	0.801
Validation Group							
Logistic Regression	0.708	0.657	0.851	0.463	0.613	0.757	0.713
Random Forest	0.734	0.686	0.699	0.672	0.681	0.691	0.69
Gradient Boosting	0.837	0.763	0.716	0.81	0.79	0.74	0.751
SVM	0.731	0.671	0.71	0.632	0.658	0.685	0.683
Neural Network	0.683	0.633	0.695	0.57	0.618	0.652	0.654
CNN	0.762	0.696	0.698	0.693	0.695	0.697	0.697
AdaBoost	0.765	0.708	0.725	0.691	0.701	0.715	0.713
Stacking Ensemble	0.867	0.794	0.749	0.84	0.824	0.77	0.785

AUC, area under the curve (receiver operating characteristic); PPV, positive predictive value; NPV, negative predictive value; SVM, support vector machine; CNN, convolutional neural network.

In the validation cohort, the Stacking Ensemble model achieved robust performance metrics: AUC of 0.867 (95% CI: 0.830–0.904), accuracy of 79.4% (95% CI: 74.2%–84.6%), sensitivity of 74.9% (95% CI: 68.5%–81.3%), specificity of 84.0% (95% CI: 79.1%–88.9%), PPV of 82.4% (95% CI: 76.8%–88.0%), NPV of 77.0% (95% CI: 71.2%–82.8%), and F1-score of 78.5%. The Gradient Boosting model also demonstrated competitive performance with an AUC of 0.837 (95% CI: 0.798–0.876), while traditional logistic regression achieved an AUC of 0.708 (95% CI: 0.662–0.754).

Considering the balance between performance metrics, model complexity, interpretability, and clinical applicability, the Stacking Ensemble approach was selected as the optimal model for subsequent clinical implementation and validation.

### Model validation and calibration

**Figure 2:**
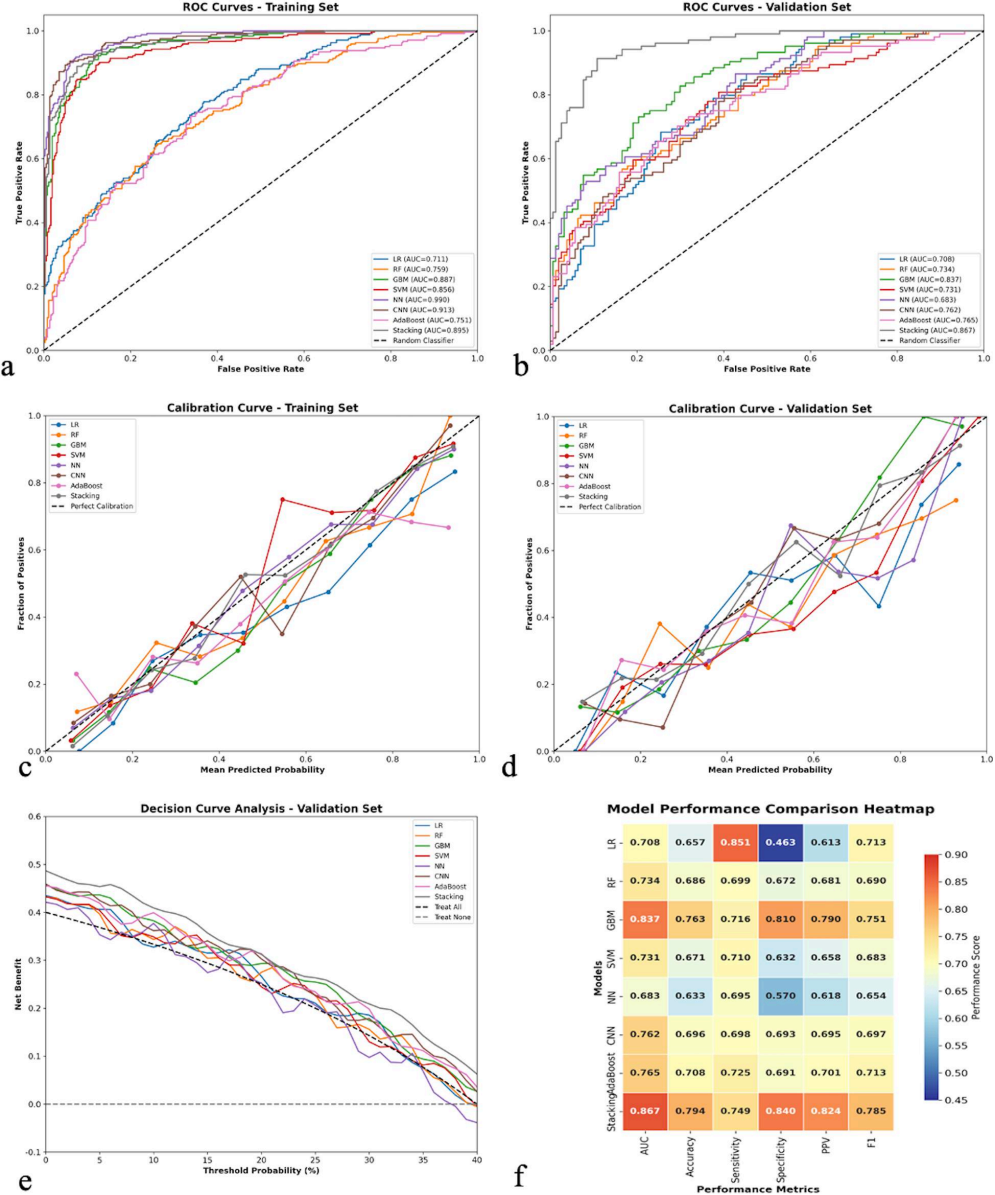
Comprehensive model performance evaluation and comparison. **(a)** Receiver operating characteristic (ROC) curves for all eight machine learning models in the training set, showing discriminative performance with area under the curve (AUC) values. **(b)** ROC curves for the validation set demonstrating model generalizability. **(c)** Calibration curves for the training set comparing predicted vs. observed probabilities, with the diagonal dashed line representing perfect calibration. **(d)** Calibration curves for the validation set. **(e)** Decision curve analysis for the validation set showing net clinical benefit across different threshold probabilities, with “Treat All” and “Treat None” strategies as references. **(f)** Model performance comparison heatmap displaying standardized performance metrics (AUC, accuracy, sensitivity, specificity, PPV, F1-score) across all models, with color intensity indicating performance levels. LR, logistic regression; RF, random forest; GBM, gradient boosting machine; SVM, support vector machine; NN, neural network; CNN, convolutional neural network; PPV, positive predictive value.

The ROC curves for both training and validation sets are presented in Figures 2a,b. The Stacking Ensemble model maintained consistent performance across both datasets, demonstrating good model stability. The calibration curves showed reasonable agreement between predicted and observed probabilities, particularly in the validation set, indicating good model calibration (Figures 2c,d).

The decision curve analysis (Figure 2e) revealed that most models provided clinical benefit across a wide range of threshold probabilities, with the Stacking Ensemble model showing superior net benefit compared to both “treat all” and “treat none” strategies. The performance comparison heatmap (Figure 2f) visualized the comprehensive performance metrics across all models, confirming the superiority of ensemble methods.

### Model interpretability analysis

**Figure 3:**
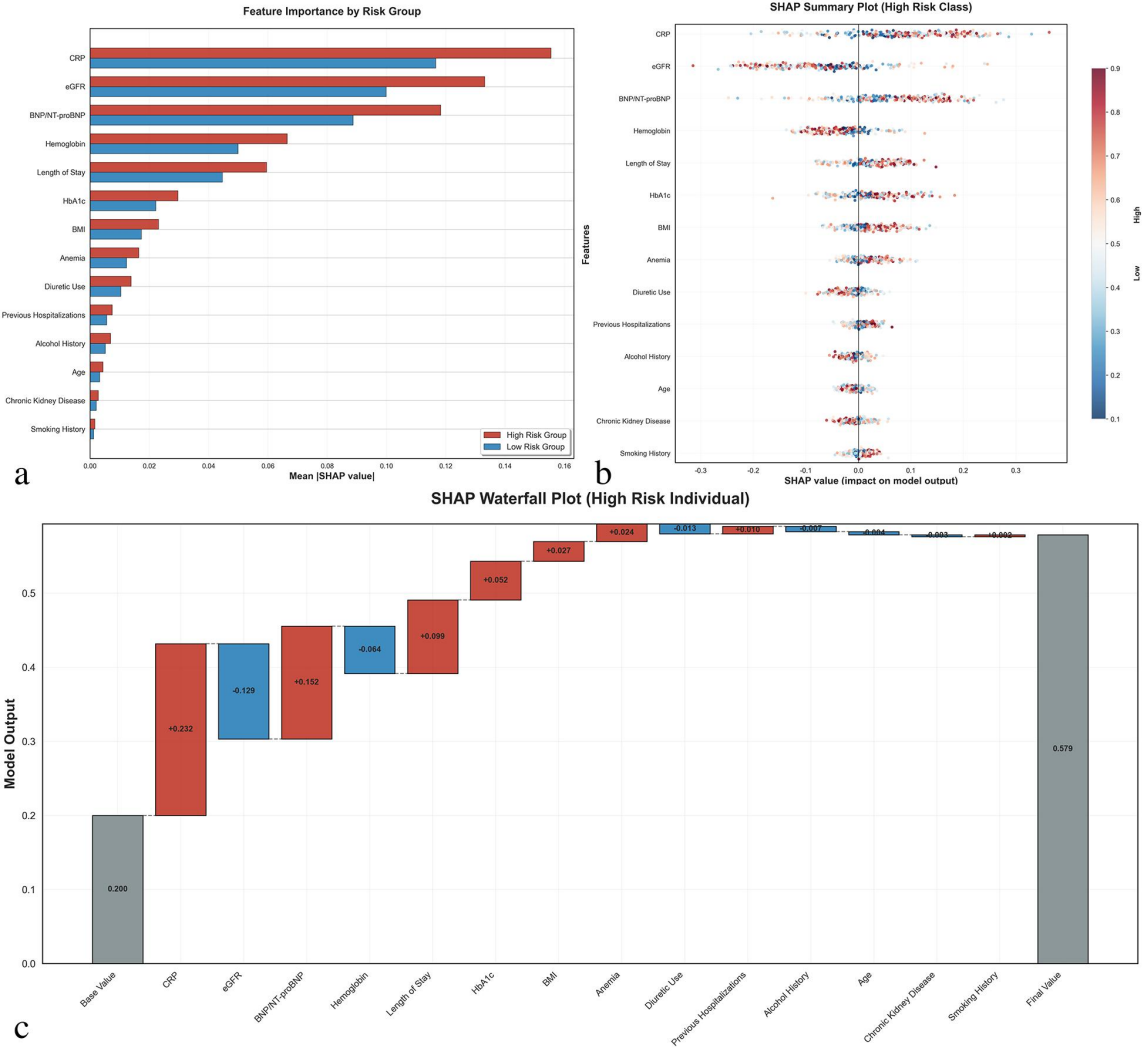
SHAP (SHapley additive exPlanations) interpretability analysis. **(a)** Feature importance comparison between high-risk and low-risk groups showing mean absolute SHAP values for the top 14 selected features. Red bars represent high-risk patients, blue bars represent low-risk patients. **(b)** SHAP summary plot for high-risk class showing the distribution of SHAP values for each feature across all patients. Each dot represents one patient, with color indicating feature value (red = high, blue = low) and *x*-axis position showing the impact on model prediction. **(c)** SHAP waterfall plot for a representative high-risk individual demonstrating how each feature contributes to the final prediction. Positive SHAP values (red) increase readmission risk, while negative values (blue) decrease risk. The base value represents the model's expected output, and the final prediction probability is shown on the right. CRP, C-reactive protein; eGFR, estimated glomerular filtration rate; BNP/NT-proBNP, B-type natriuretic peptide/N-terminal pro-B-type natriuretic peptide; HbA1c, hemoglobin A1c; BMI, body mass index.

SHAP analysis was conducted to enhance model interpretability (Figure 3). The feature importance ranking revealed that CRP was the most influential predictor, followed by eGFR, BNP/NT-proBNP, hemoglobin, and length of hospital stay. The SHAP summary plot demonstrated that higher CRP levels, lower eGFR, elevated BNP/NT-proBNP, lower hemoglobin levels, and longer hospital stays were associated with increased readmission risk.

The SHAP waterfall plot for a high-risk individual illustrated how each feature contributed to the final prediction, with CRP (+0.271), eGFR (−0.132), and BNP/NT-proBNP (+0.152) being the most significant contributors. This analysis provides clinicians with transparent insights into the decision-making process of the prediction model.

### Clinical risk calculator development

**Figure 4:**
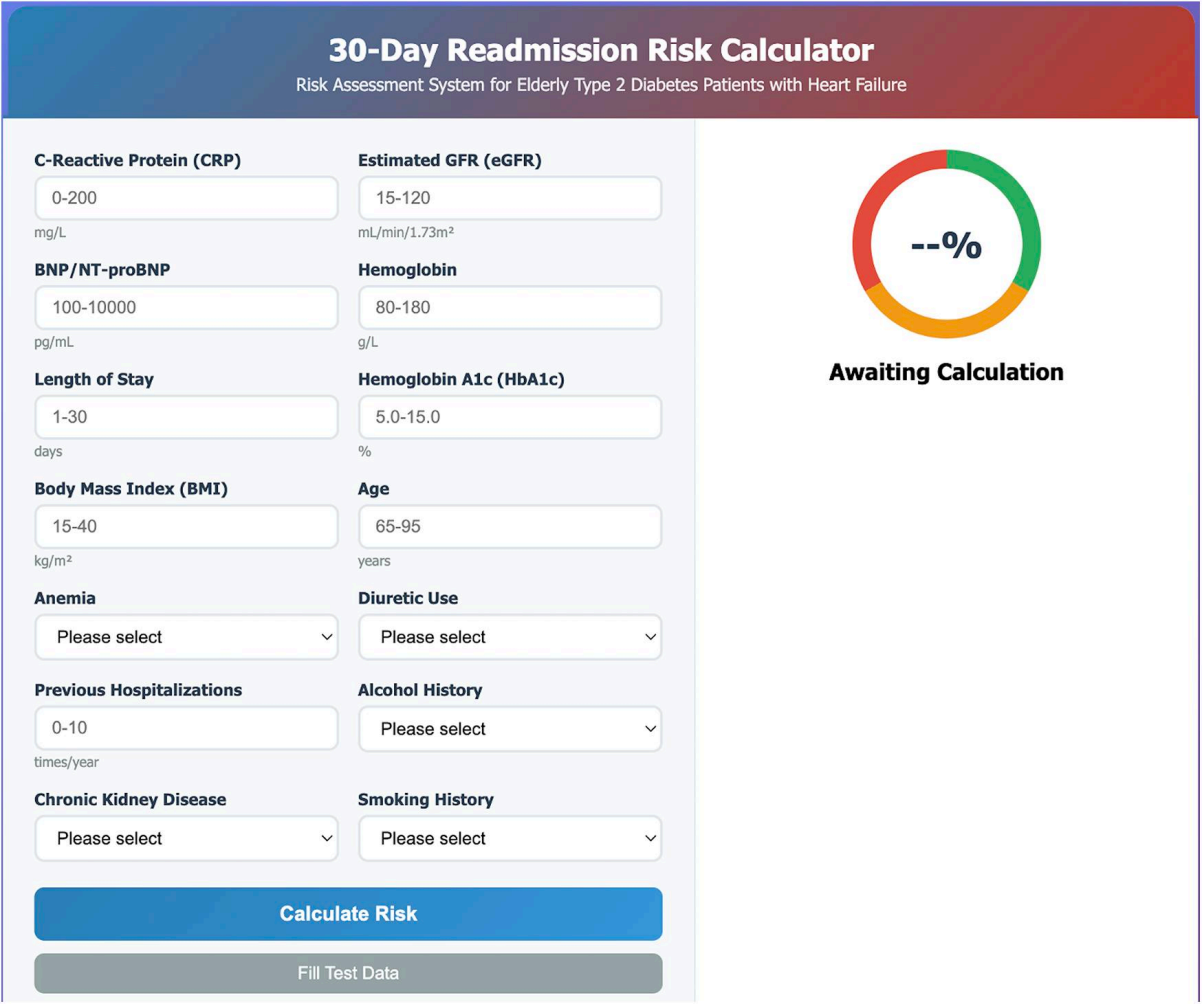
Web-based clinical risk calculator interface. Screenshot of the user-friendly 30-day readmission risk calculator developed for point-of-care clinical decision-making. The interface includes input fields for all 14 selected predictive features: laboratory parameters (CRP, eGFR, BNP/NT-proBNP, hemoglobin, HbA1c), clinical characteristics (length of stay, BMI, age, previous hospitalizations), and categorical variables (anemia, diuretic use, alcohol history, chronic kidney disease, smoking history). The right panel displays a color-coded risk gauge with real-time probability calculation, ranging from low risk (green) to high risk (red). The calculator provides immediate risk assessment with visual feedback to facilitate clinical interpretation and intervention planning.

Based on the optimal model performance, a user-friendly web-based clinical risk calculator was developed (Figure 4). The calculator incorporates the 14 selected features and provides real-time risk assessment with a visual risk gauge ranging from low (green) to high (red) risk categories. The tool includes input validation and provides immediate feedback to clinicians, facilitating point-of-care decision-making.

The calculator interface allows healthcare providers to input patient-specific data including laboratory values, clinical parameters, and medical history. Upon calculation, the tool displays the predicted 30-day readmission probability as both a percentage and a color-coded risk level, enabling quick clinical interpretation and appropriate intervention planning.

## Discussion

This study successfully developed and validated a machine learning-based prediction model for 30-day readmission risk in elderly patients with T2DM complicated by HF. The Stacking Ensemble model demonstrated superior performance with an AUC of 0.867 in the validation cohort, indicating good discriminative ability for identifying high-risk patients. Our findings contribute to the growing body of evidence supporting the use of advanced machine learning techniques in clinical risk prediction, particularly for complex patient populations with multiple comorbidities (17).

The 12.4% aggregate 30-day readmission frequency documented in our investigation falls within the range reported in contemporary literature (10%–25%) for comparable patient cohorts (5–7). This rate underscores the significant healthcare burden associated with this vulnerable population and highlights the critical need for effective risk stratification tools.

Our algorithm determined 14 essential predictors, with C-reactive protein, estimated glomerular filtration rate, and B-type natriuretic peptide/N-terminal pro-B-type natriuretic peptide appearing as the most significant factors based on SHAP evaluation. These findings are consistent with recent studies emphasizing the importance of inflammatory markers and cardiac biomarkers in predicting adverse outcomes in heart failure patients (18, 19).

The significance of C-reactive protein as the primary predictor corresponds with accumulating evidence regarding inflammation's contribution to cardiac failure advancement and readmission probability (20, 21). This finding challenges current discharge practices that primarily focus on hemodynamic stabilization and symptom resolution. Clinicians should consider incorporating CRP monitoring into discharge readiness criteria, potentially delaying discharge for patients with persistently elevated CRP despite clinical stability. This represents a shift from symptom-based to biomarker-guided discharge decision-making. Increased C-reactive protein concentrations indicate systemic inflammatory processes, which have been linked to adverse outcomes in both diabetic and cardiac failure patient groups (22). Recent studies have shown that anti-inflammatory interventions may improve outcomes in heart failure patients, suggesting potential therapeutic targets for high-risk individuals (23).

The substantial contribution of estimated glomerular filtration rate to our algorithm mirrors the well-documented cardiorenal syndrome occurring in patients with simultaneous diabetes and cardiac failure (24, 25). Our model reveals that the interaction between eGFR and BNP/NT-proBNP creates synergistic risk that exceeds either factor alone, suggesting that current fragmented care models—where nephrology, cardiology, and endocrinology manage conditions separately—may be inadequate. This supports developing integrated cardiorenal-metabolic clinics with coordinated medication optimization strategies. Declining kidney function serves as both an outcome and catalyst for cardiac failure advancement, establishing a detrimental cycle that elevates readmission probability (26). Recent guidelines emphasize the importance of renal function monitoring and optimization in this population (2).

B-type natriuretic peptide/N-terminal pro-B-type natriuretic peptide concentrations, recognized markers of cardiac strain and fluid overload, have repeatedly been correlated with unfavorable outcomes in cardiac failure patients (27, 28). The inclusion of these biomarkers in our model reinforces their clinical utility and supports current guideline recommendations for their use in heart failure management (29).

Contemporary systematic evaluations have emphasized the difficulties in creating broadly applicable readmission prediction algorithms, with the majority attaining moderate discriminative capacity (30). To contextualize our findings, we compared our model with established clinical tools. The LACE index achieves AUC values of 0.61–0.68 in heart failure populations (31), while the HOSPITAL score demonstrates AUC ranges of 0.65–0.72 (32). Recent machine learning studies in heart failure readmission prediction report AUC values of 0.72–0.79 (33, 34). Our Stacking Ensemble model's AUC of 0.867 represents a substantial improvement of 0.09–0.26 over these tools, likely reflecting our model's ability to capture complex non-linear interactions through ensemble learning that traditional scoring systems and single algorithm approaches cannot adequately address in elderly patients with multiple comorbidities. Our study advances the field by demonstrating that ensemble machine learning approaches can achieve clinically meaningful performance in this complex patient population.

An important observation in our study was the substantial performance drop in highly complex models between training and validation sets. The Neural Network achieved an AUC of 0.990 in training but dropped to 0.683 in validation, indicating significant overfitting despite regularization techniques. Similarly, the CNN model showed reduced generalizability (training AUC: 0.913 vs. validation AUC: 0.762). In contrast, the Stacking Ensemble demonstrated consistent performance across both cohorts (training AUC: 0.895, validation AUC: 0.867), with only a modest decrease of 0.028 compared to the Neural Network's dramatic drop of 0.307. We selected the Stacking Ensemble as our final model based on this superior balance between discriminative performance and generalizability, along with its stability and practical implementability in clinical settings. This decision prioritizes robust real-world performance over maximizing training metrics, which is essential for developing clinically reliable prediction tools.

The integration of SHAP methodology improves the clinical utility of our algorithm by delivering transparent, comprehensible insights into individual patient risk determinants. This interpretability is crucial for clinical acceptance and implementation, as healthcare providers need to understand the rationale behind risk predictions to make informed decisions.

The creation of an accessible web-based computational tool constitutes a meaningful advancement toward practical clinical deployment. Beyond prediction, our model enables risk-stratified resource allocation: high-risk patients (predicted probability >40%) could receive intensive transitional care with home monitoring and nurse coordination, while low-risk patients (<15%) receive standard discharge planning, optimizing resource utilization. However, successful implementation requires seamless integration into electronic health records with automated risk calculation and actionable care pathway recommendations to avoid alert fatigue. Studies have shown that point-of-care risk assessment tools can improve clinical decision-making and patient outcomes when properly integrated into clinical workflows. Importantly, the model should augment rather than replace clinical judgment, serving as a framework for shared decision-making that incorporates both quantitative risk and patient-specific factors including social support and medication adherence capacity.

Multiple advantages differentiate our investigation from prior research efforts. Initially, the emphasis on aged patients with combined pathology addresses a notable void in the existing literature, as most studies examine single conditions. Additionally, the thorough assessment of various artificial intelligence algorithms with stringent validation methodologies strengthens the dependability of our results. Third, the inclusion of both clinical and laboratory variables provides a holistic assessment of patient risk.

Our study cohort comprised 56.1% female patients, which is higher than some reported cohorts but consistent with the epidemiology of heart failure in elderly populations. This sex distribution reflects several well-established demographic patterns. Women have longer life expectancy and constitute a larger proportion of the very elderly population, where heart failure prevalence is highest (35, 36). Additionally, in patients aged ≥75 years, the proportion of female heart failure patients increases substantially, with women more commonly presenting with heart failure with preserved ejection fraction and diabetes-related cardiac dysfunction (37). During feature selection, sex was evaluated but not retained as an independent predictor of 30-day readmission, suggesting that after accounting for clinical and laboratory variables, sex itself did not directly influence readmission risk in our model. However, the higher proportion of female patients may affect model generalizability to populations with different sex distributions, warranting external validation in more diverse cohorts.

Notwithstanding these advantages, multiple limitations warrant recognition. First, the retrospective single-center design represents a significant limitation. Our tertiary care center may attract more complex patients, potentially inflating readmission rates and risk estimates. The exclusion criteria, particularly for patients with missing data >20% and in-hospital deaths, may have selected a healthier cohort, leading to underestimation of true readmission risk. Additionally, our data (2020–2023) spans the COVID-19 pandemic era, which may have influenced hospitalization patterns and limit applicability to other periods. Our patient population reflects the specific demographic characteristics, clinical practices, treatment protocols, and healthcare resources of a single tertiary care center in China, which may differ substantially from other institutions regionally and internationally. Geographic variation in disease prevalence, treatment patterns, medication availability, and healthcare delivery models could affect model performance when applied to different populations. Second, the absence of temporal validation is critical. Our training and validation cohorts were randomly split from the same period rather than sequentially validated on future data, which does not account for temporal drift in clinical practices, evolving treatment guidelines (e.g., increased SGLT2 inhibitor use), or changing patient characteristics that could affect model calibration over time. Third, our model relies on discharge data only, potentially missing important longitudinal trajectories, post-discharge factors, and social determinants of health (housing stability, caregiver support, socioeconomic status) known to impact readmission risk. Additionally, unmeasured confounders such as frailty, cognitive function, and health literacy—increasingly recognized as important readmission predictors—were not systematically captured in our records. Furthermore, the higher proportion of female patients (56.1%), while consistent with elderly heart failure demographics, may limit generalizability to populations with different sex distributions. Subsequent prospective investigations with external validation in multiple independent centers across diverse geographic regions and healthcare systems are essential to establish the model's generalizability and clinical utility in broader populations. Future studies should implement temporal validation designs and randomized controlled trials to assess whether model-guided interventions actually reduce readmission rates.

Our findings have important clinical implications, as healthcare providers can use this model to identify high-risk patients and implement targeted interventions, including intensive discharge planning, enhanced follow-up, and transitional care programs.

This investigation successfully constructed and verified a high-performance artificial intelligence algorithm for forecasting 30-day readmission probability in aged T2DM patients with cardiac failure. The algorithm's excellent discriminative capacity, coupled with its interpretability and clinical accessibility, establishes it as a beneficial instrument for enhancing patient management and decreasing healthcare expenditures. Subsequent research endeavors should concentrate on prospective verification and deployment studies to establish the algorithm's influence on clinical results.

## Data Availability

The raw data supporting the conclusions of this article will be made available by the authors, without undue reservation.
